# Hesperetin alleviates cerebral ischemia–reperfusion injury by suppressing neuronal ferroptosis

**DOI:** 10.1038/s41598-026-51280-w

**Published:** 2026-05-05

**Authors:** Yuhao Xue, Huacheng Wang, Qian Cheng, Jinlong Wan, Xueqing Song, Chunyang Huang, Xinming Huang, Qingchun Mu, Yufei Zhang

**Affiliations:** 1 Basic Medical College, Guilin Medical University, Guilin, 541199 China; 2 School of Biomedical Engineering, Anhui Medical University, Hefei, 230022 China; 3 Gaozhou People’s Hospital, Guangdong Medical University, Maoming, 525200 China; 4 Mudanjiang Medical University, Mudanjiang, 157011 China; 5 School of Pharmacy, Guilin Medical University, Guilin, 541199 China; 6 Key Laboratory of Cell and Gene Therapy for Regional High-incidence Diseases,Education Department of Guangxi Zhuang Autonomous Region, Guilin Medical University, Guilin, 541199 China

**Keywords:** Hesperetin, Ischemia–reperfusion injury, Ferroptosis, Apoptosis

## Abstract

Hesperetin (HSP), a natural flavonoid, demonstrates significant therapeutic effects on cardiovascular and cerebrovascular diseases and displays a strong application potential in the aspects of anti-inflammation and anti-oxidation. Cerebral ischemia–reperfusion is accompanied by the generation of inflammatory storms and the accumulation of reactive oxygen species (ROS), ultimately resulting in neuronal damage. While Hesperetin has been widely studied in the treatment of cardiovascular diseases, its potential for treating cerebral ischemia–reperfusion injury remains underexplored. This study aimed to discuss the potential protective mechanism of HSP on cerebral ischemia–reperfusion injury (CIRI). Ferroptosis, a form of cell death driven by iron-dependent phospholipid peroxidation is associated with neuronal damage during cerebral ischemia and subsequent reperfusion injury. Our findings indicate that HSP can confer neuronal protection after CIRI by inhibiting neuronal ferroptosis. Specifically, HSP could significantly up-regulate glutathione (GSH) levels, and up-regulate glutathione peroxidase 4(GPX4) after CIRI, thereby inhibiting cell ferroptosis. Furthermore, we observed a significant reduction in lipid peroxidation products and ROS levels, we have also obtained the same results in vivo animal experiments. In conclusion, HSP played a protective role in CIRI by regulating intracellular iron ions levels, as well as GSH and GPX4 contents to inhibit neuronal ferroptosis after CIRI.

## Introduction

With the aging population, cardiovascular and cerebrovascular diseases have emerged as significant threats to human health and survival, Among these, stroke is the second largest leading cause of death worldwide, with approximately 80% of stroke cases are classified as ischemic stroke^1^. Prompt thrombolysis is a crucial intervention to reduce the severity of cerebral infarction after ischemic stroke. However, a series of stress responses induced by thrombolysis can further induce neuronal death, known as cerebral ischemia–reperfusion injury^2,3^. This type of injury is characterized by high rates of morbidity, disability and mortality^4^. Although the underlying mechanisms of cerebral ischemia–reperfusion injury is still unclear, the existing research suggests that it involves factors such as oxidative stress, inflammatory reaction, calcium ion overload, excitatory toxicity, apoptosis^5,6^.

Ferroptosis is a regulated, iron-dependent form of non-apoptotic cell death driven by lipid peroxidation. It is characterized by down-regulation of GPX4, accumulation of intracellular ROS, and dysregulation of intracellular lipid peroxides. This process can be induced or inhibited through pharmacological and genetic perturbations^7,8^. Studies have demonstrated that ferroptosis plays a critical role in cerebral ischemia–reperfusion injury, providing a potential target for the treatment of this condition ^9,10^.

**Scheme 1:**
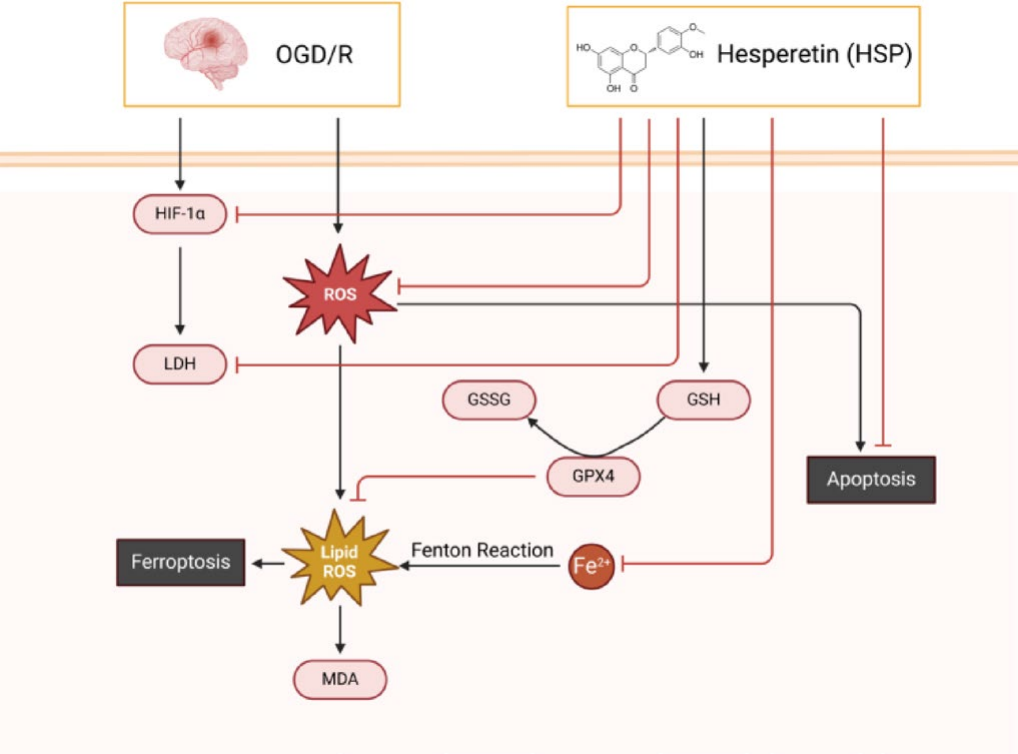
HSP inhibits cerebral ischemia–reperfusion injury by suppressing neuronal ferroptosis and apoptosis. (Created in BioRender.com).

Hesperetin (HSP) is a flavonoid compound derived from citrus, known for its significant anti-inflammatory, anti-oxidant and anti-apoptotic effects^11–13^, studies have shown that HSP exerts neuroprotective effects by reducing neuroinflammation and oxidative stress^12,14,15^. These findings suggest that HSP has promising protective properties effects against nerve injury. The propose of this study was to investigate the protective effect and potential therapeutic mechanism of HSP on nerve injury after cerebral ischemia–reperfusion injury both in vitro and in vivo experiments (Scheme 1), thereby enhancing the potential for HSP to be developed as a therapeutic agent for cerebral ischemia–reperfusion injury.

## Results

### In vitro experiment

#### Effect of HSP at different concentrations on PC12 cells after OGD/R treatment

**Fig. 1:**
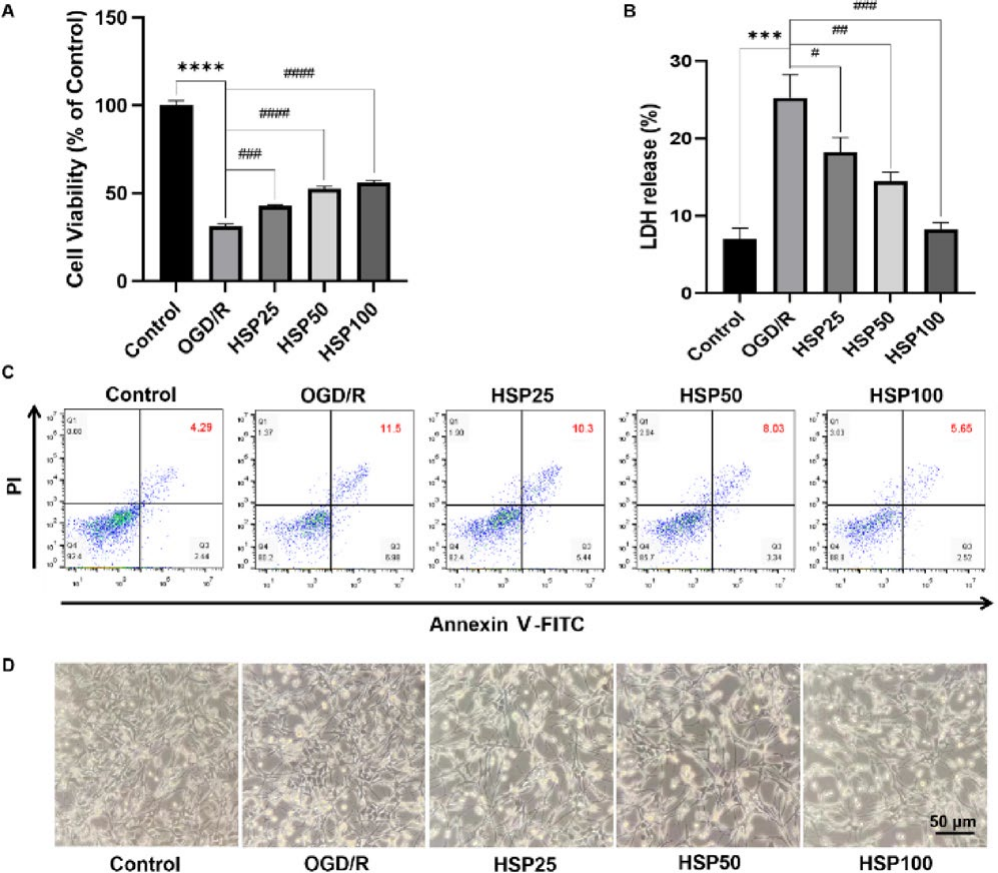
Effect of HSP on cell viability after OGD/R. (**A**) CCK-8 method for cell viability, (**B**) cell LDH content determination, and (**C**) flow cytometry for apoptosis, (**D**) cellular morphology.

The results of CCK-8 assay showed that the cell viability was significantly decreased by 31.4% after OGD/R compared to the control group. However, treatment with 100 μmol of HSP increased cell viability to 56.1% (Fig. 1A). Redox homeostasis is essential for the normal life activities of cells and lipid peroxidation serves as an important regulatory factor in determining cell fate, as well as a significant indicator of ferroptosis^16^. Lactate dehydrogenase (LDH), an important enzyme in the anaerobic glycolysis of glucose, reflects the degree of cellular hypoxia. As shown in Fig. 1B, the corresponding kit was used to measure the release of LDH to evaluate the cell viability. Compared to the control group, ischemia–reperfusion significantly elevated the LDH level in cells, and this increase was significantly reversed by HSP in a dose-dependent manner.

To further verify the effect of HSP on cell survival following OGD/R, flow cytometry analysis revealed that the apoptotic rate was significantly increased after OGD/R compared to the control group (Fig. 1C). After treatment with 100 μmol of HSP, the apoptotic rate was reduced to 5.65%, which was close to the apoptotic rate in the control group. Additionally, morphological changes in PC12 cells and the disappearance of synapses after OGD/R were observed under a phase contrast microscope, while HSP treatment maintained the stability of cell morphology (Fig. 1D).

#### HSP treatment reduces OGD/R-induced oxidative stress injury

**Fig. 2:**
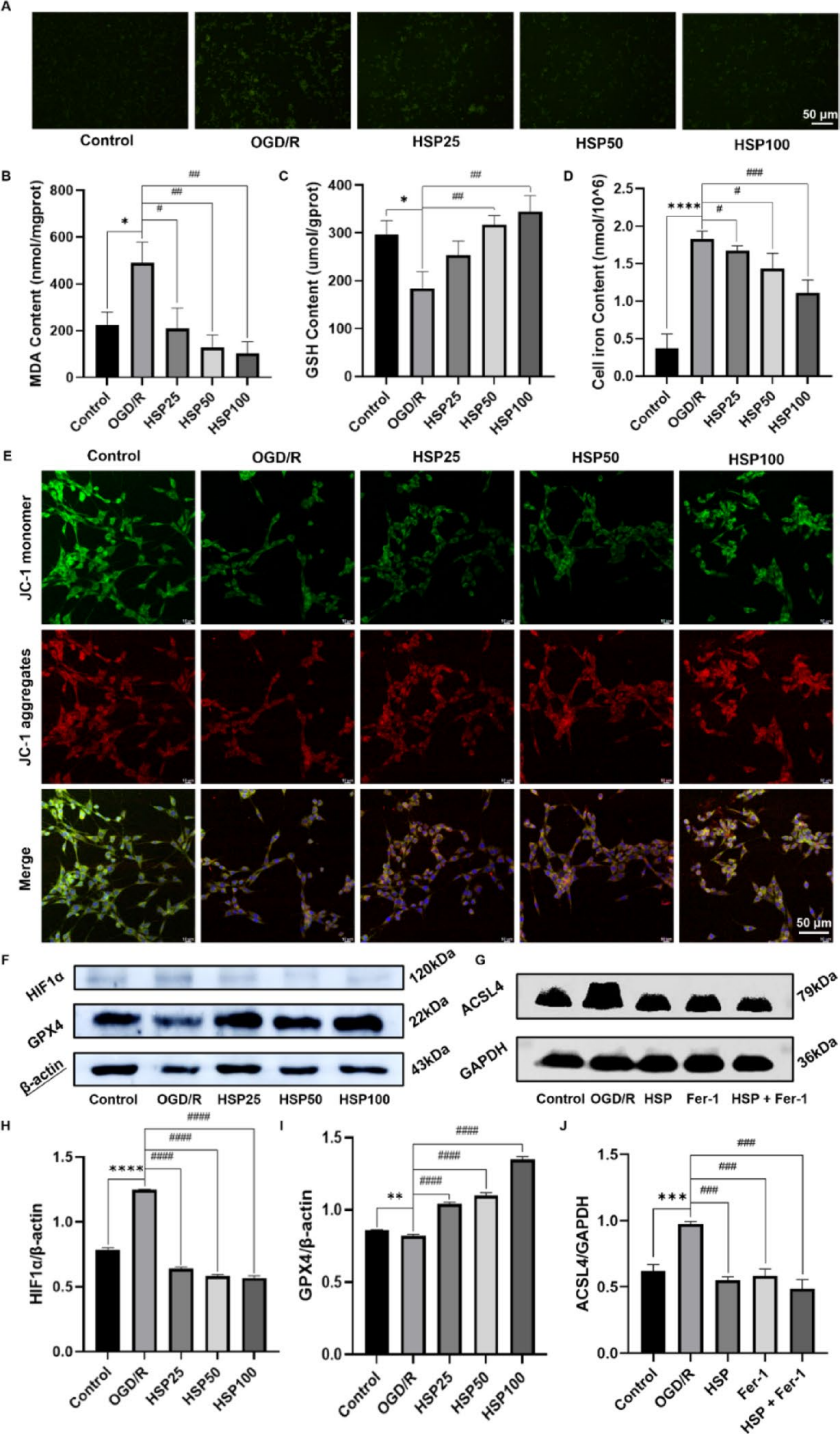
Evaluation of in vitro antioxidant activity of HSP. (**A**) laser scanning confocal microscope is used to determine ROS clearance rate, (**B**) MDA content determination, (**C**) GSH content determination, (**D**) intracellular iron ion content quantification, (**E**) laser scanning confocal microscope is used to observe mitochondrial membrane potential, (**F**) Western blot results of HIF1α and VEGF, (**G**) Western blot results of ACSL4, (**H**-**I**) quantitative analysis of HIF1α, VEGF and ACSL4.

Oxidative stress, characterized by the accumulation of ROS and malondialdehyde(MDA), the consumption of GSH, and alterations in mitochondrial membrane potential, is a significant feature of post-ischemic reperfusion^17^. The cellular ROS content was measured by immunofluorescence as shown in Fig. 2A. After OGD/R treatment, there was a marked accumulation of ROS in the cells, HSP treatment inhibited ROS accumulation in a dose-dependent manner, at the concentration of HSP was 100 μmol, ROS content was significantly reduced.

MDA, the final decomposition product of membrane lipid peroxidation, serves as an indicator of oxidative lipid damage. Under OGD/R conditions, MDA levels were significantly increased, whereas the HSP (25 µM) treatment significantly decreased the levels of MDA to the normal (Fig. 2B), indicating that HSP effectively inhibits lipid peroxidation.

Glutathione (GSH), a prevalent tripeptidyl molecule, plays a crucial role in protecting cells from oxidative stress-induced cellular damage as well as facilitating drug metabolism. Decreased GSH levels are associated with the common features of cerebral ischemia–reperfusion injury. As demonstrated in Fig. 2C, the GSH levels decreased significantly following OGD/R treatment compared to the control group, and treatment with HSP significantly increased GSH levels compared to the OGD/R group. showing that HSP effectively enhances anti-oxidant capacity.

Iron is an essential trace element in the human body, typically bound to transferrin as trivalent iron ions. Excessive intracellular iron can lead to the generation of free radicals, resulting in lipid peroxidation, DNA damage, and protein damage. Consequently, iron accumulation is a classical mechanism regulating ferroptosis. As shown in Fig. 2D, intracellular iron content significantly increased after OGD/R. With increasing doses of HSP, intracellular iron levels decreased in a dose-dependent manner, indicating that HSP effectively regulates intracellular iron levels to normal and blocks ferroptosis induced by iron overload.

Mitochondria are vital organelles responsible for ATP production, and maintaining a stable mitochondrial membrane potential is essential for oxidative phosphorylation and overall mitochondrial function. A decline in mitochondrial membrane potential is a hallmark of early apoptosis. Cells subjected to OGD/R were treated with varying concentrations of HSP and stained with JC-1 dye. Observations under a confocal microscope revealed that red fluorescence diminished after OGD/R but gradually intensified following HSP treatment. At 100 μM, the red fluorescence intensity was comparable to that of the normal group, significantly inhibiting the decline in mitochondrial membrane potential after OGD/R (Fig. 2E).

Hypoxia-inducible factor 1-alpha (HIF1α) is a critical marker of cellular hypoxia and is significantly upregulated following cerebral ischemia/reperfusion. To verify this, we employed Western blot analysis, which demonstrated that HSP significantly reduced HIF1α expression in a dose-dependent manner after cerebral ischemia–reperfusion. Glutathione peroxidase 4 (GPX4) is regarded as a key resistance factor against ferroptosis, functioning as an antioxidant by catalyzing the reduction of lipid peroxides. Our experimental results indicated that GPX4 expression was downregulated following cerebral ischemia–reperfusion compared to the normal group. However, treatment with varying doses of HSP led to a significant increase in GPX4 expression, surpassing levels observed in the normal group. These findings suggest that HSP can upregulate GPX4 expression after OGD/R in a dose-dependent manner, thereby inhibiting the occurrence of ferroptosis (Fig. 2F, H, I).

ACSL4 promotes the synthesis of unsaturated fatty acids and enhances lipid peroxidation, making it a key driver of ferroptosis. Western blot was used to verify whether HSP regulates ACSL4 following cellular OGD/R, as well as whether HSP regulates ACSL4 in conjunction with Fer-1. Fer-1 is a ferroptosis inhibitor that suppresses ferroptosis by inhibiting lipid peroxidation. Studies have shown that ACSL4 is significantly upregulated following OGD/R, while both HSP and Fer-1 significantly downregulate ACSL4 expression following OGD/R, and the effects of HSP and Fer-1 are comparable (Fig. 2G, J).

#### HSP attenuates cerebral ischemia–reperfusion injury in mice

**Fig. 3:**
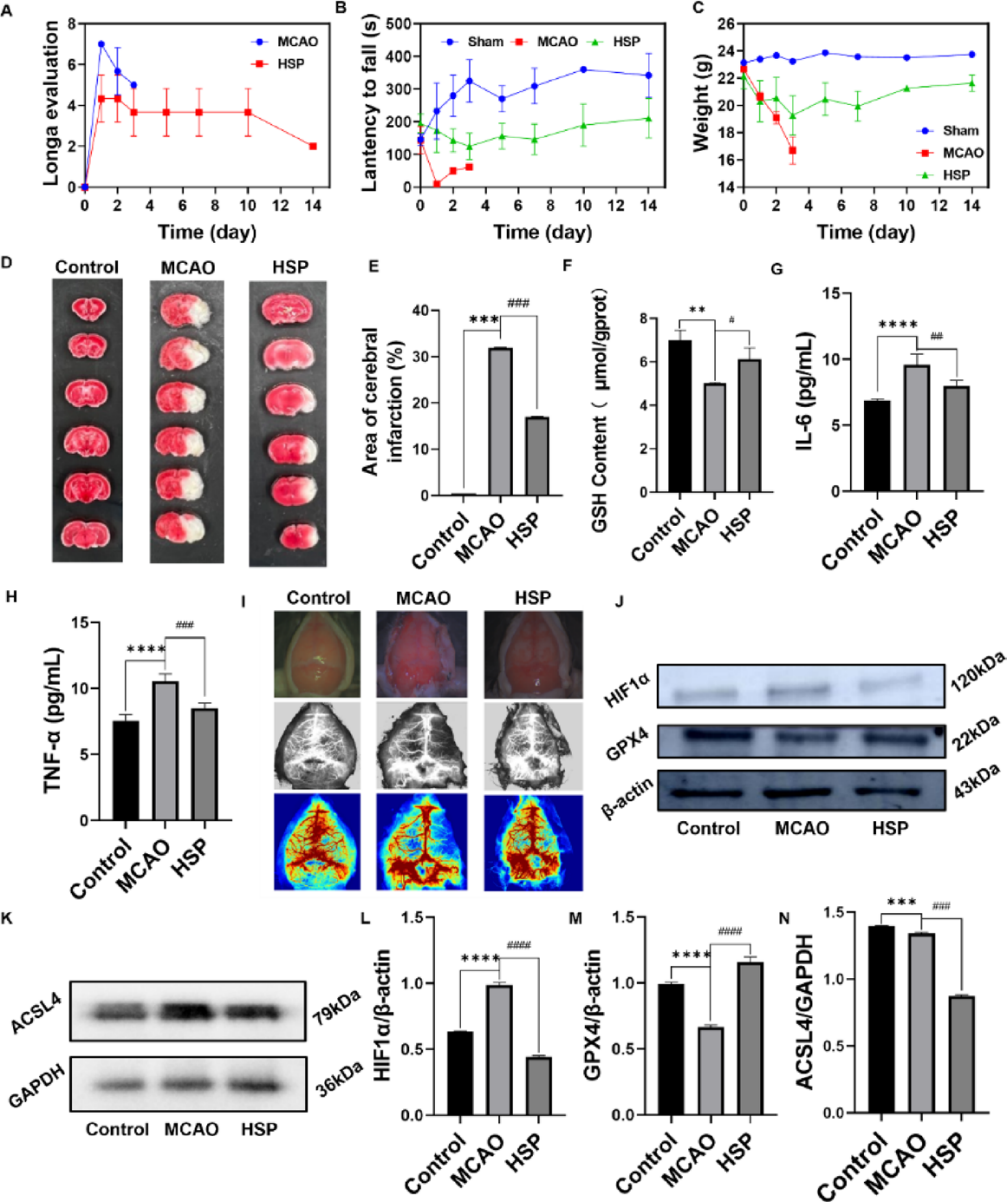
To verify the neuroprotective effect of HSP in vivo. (**A**) mice behavioral score, (**B**) mice rotarod test, (**C**) mice body weight monitoring, (**D**, **E**) TTC staining of brain sections and infarct volume quantification, (**F**) determination of GSH content in brain tissue, (**G**) IL-6 expression in the brain tissue of mice in each group, (H) TNF-α expression in the brain tissue of mice in each group, (**I**) LSCI imaging results at day 3 in each group of mice, (**J**) Western blot results of HIF1α and VEGF, (**K**) Western blot results of ACSL4, (**L**-**N**) quantitative analysis of HIF1α, VEGF and ACSL4.

HSP treatment could significantly improve the neurological scores of mice (Fig. 3A). The rotarod test results showed that the rotarod falling time was significantly decreased in the MCAO group on the first day after operation. The rotarod falling time was increased on the second and third days after operation, but the mice died one after another on the fourth day after operation. Compared with the HSP group, the rotarod falling time was slightly decreased on the first three days after operation, and began to rise three days later, and basically returned to the normal level on the fourteenth day after operation (Fig. 3B). Compared with Sham group, the body weight of mice in MCAO group showed a significant downward trend after operation and all died on the 4rd day after operation, The body weight of mice in HSP group began to increase on the 3rd day after operation, and reached the normal level on the 14th day after operation (Fig. 3C). In order to observe the therapeutic effect of HSP on cerebral ischemia–reperfusion injury more intuitively, we carried out TTC staining on the back of the brain of mice. The experiment results showed that the untreated mice there was a large infarction in the brain with the infarct area reaching about 32%. However, after HSP treatment, the cerebral infarct volume of mice was significantly reduced to about 16%, which provided macro data support for further verification of the mechanism of HSP treatment of cerebral ischemia–reperfusion injury (Fig. 3D, E).

To further verify the mechanism of HSP on cerebral ischemia–reperfusion injury in vivo, we took brain homogenates from different groups of mice and extracted the proteins for GSH content determination and western blot analysis. Interestingly, the in vivo experimental results were consistent with the in vitro verification results. The results showed that after MCAO, the GSH consumption in mouse brain tissue was decreased, and strong oxidative stress reaction occurred in mice after MCAO (Fig. 3F). The brain tissues of mice in each group were taken and tested by ELISA. Compared with the MCAO group, the expression levels of inflammatory factors IL-6 and TNF-α in the brain tissues of mice in the HSP group were significantly decreased and close to those in the Control group (Fig. 3G, H).

The results of LSCI assay showed that 3 d after modeling, the relative blood flow of local microcirculation in the brain of mice in the HSP group was significantly increased compared with that in the MCAO group (Fig. 3I). HIF1α is the most important regulatory factor in hypoxia environment. western blot analysis results showed that after MCAO, the protein level of HIF1α was significantly increased in mice, indicating that the mice MCAO model was successfully established. Under the action of HSP, the protein level of HIF1α was significantly decreased, indicating that HSP could significantly inhibit oxidative stress level in mice after MCAO (Fig. 3J, L).

Correspondingly, GPX4 protein level, as a marker protein of ferroptosis, has been widely used to verify the interaction between drugs and ferroptosis. The experimental results showed that the expression level of GPX4 protein in the brain tissue of MCAO mice was significantly decreased, and lipid peroxide was dysfunctional. After HSP treatment, the expression level of GPX4 protein in the brain tissue of MCAO mice was significantly increased (Fig. 3J, M), indicating that the level of lipid peroxide tended to be normal. HSP plays an important regulatory role in GPX4 after cerebral ischemia reperfusion. HSP can significantly inhibit the accumulation of lipid peroxide in the brain tissue of MCAO mice.

ACSL4 is a key molecule that regulates ferroptosis, it participates in the initiation and amplification of lipid peroxidation, thereby driving iron-dependent cell death. Its expression levels are closely associated with a cell’s susceptibility to ferroptosis. Following MCAO in mice, ACSL4 is highly expressed in brain tissue, after HSP treatment, ACSL4 expression was downregulated to lower levels (Fig. 3K, N).

#### HSP inhibits the development of ferroptosis after MCAO

**Fig. 4:**
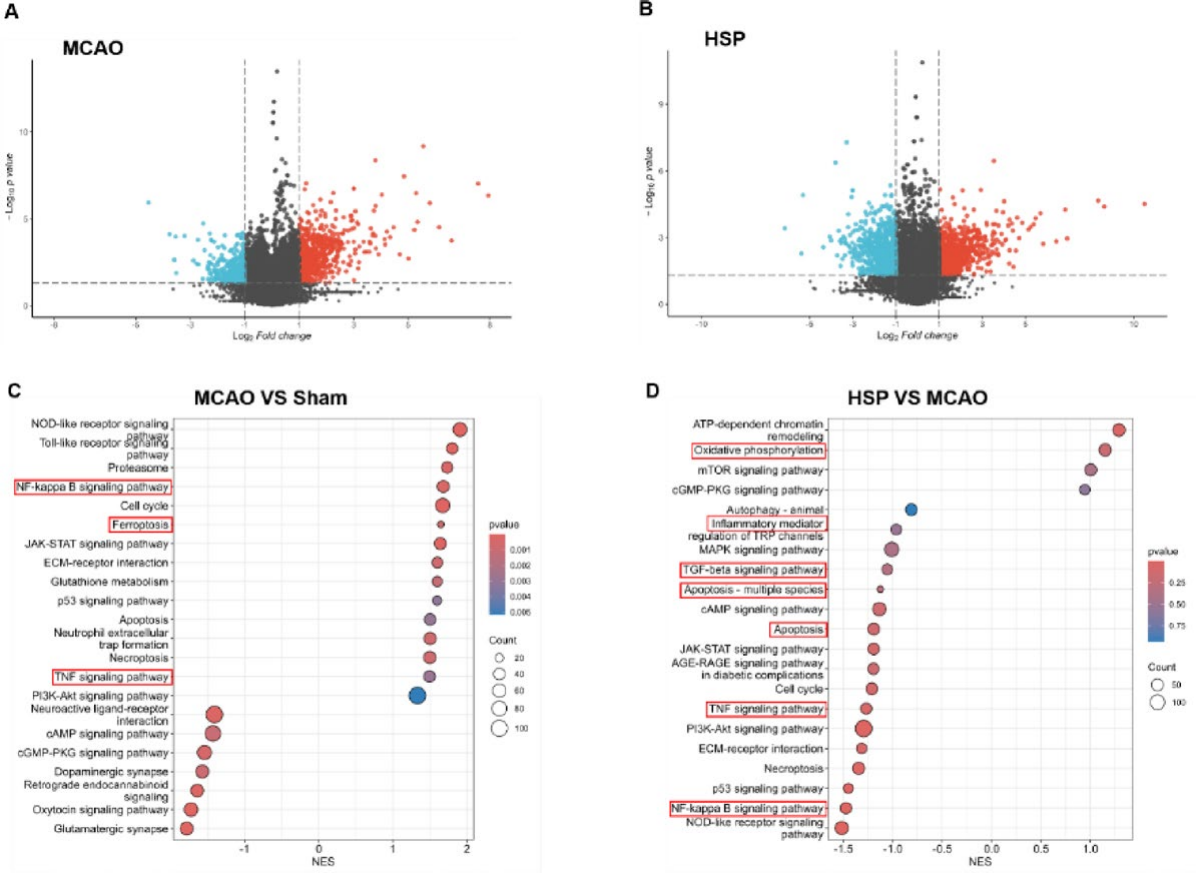
Transcriptomics analysis of HSP in mice brain after MCAO. (**A**, **B**) Intergroup differential gene analysis, (**C**, **D**) KEGG enrichment analysis.

In order to in-depth understand the regulatory effect of HSP on cerebral ischemia–reperfusion, we performed RNA sequencing analysis on mouse brain tissue. The volcanic diagram showed that compared with Sham group, MCAO group had a total of 13,822 gene expressions, 610 genes in the MCAO group were down-regulated and 949 genes were up-regulated (Fig. 4A). In addition, HSP group and Sham group had a total of 13,671 gene expressions, and HSP group showed 1781 differentially expressed genes, with 821 genes down-regulated and 960 genes up-regulated (Fig. 4B). Through GSEA enrichment analysis, it was found that both the MCAO group and the HSP group had significantly expressed genes which were mainly enriched in autophagy, immunity, inflammation, apoptosis, cell cycle energy metabolism and oxidative stress (Fig. 4C). Of note, the significant difference between the Sham group and the highly enriched ferroptosis genes in the MCAO group disappeared after HSP treatment (Fig. 4D). Therefore, based on cellular and animal experiments, we believe that HSP can reverse the cell ferroptosis induced by ischemic-reperfusion injury, by regulating the lipid peroxidation homeostasis after MCAO.

#### Hesperetin-GPX4 molecular docking

Through the target-related proteins in the PDB database and the treatment of proteins and ligands, a reasonable active cavity box model was established and then molecular docking was performed with the compound hesperetin. The results were displayed by pymol (version 2.3), LigPlot + (v.2.2.8).

**Fig. 5:**
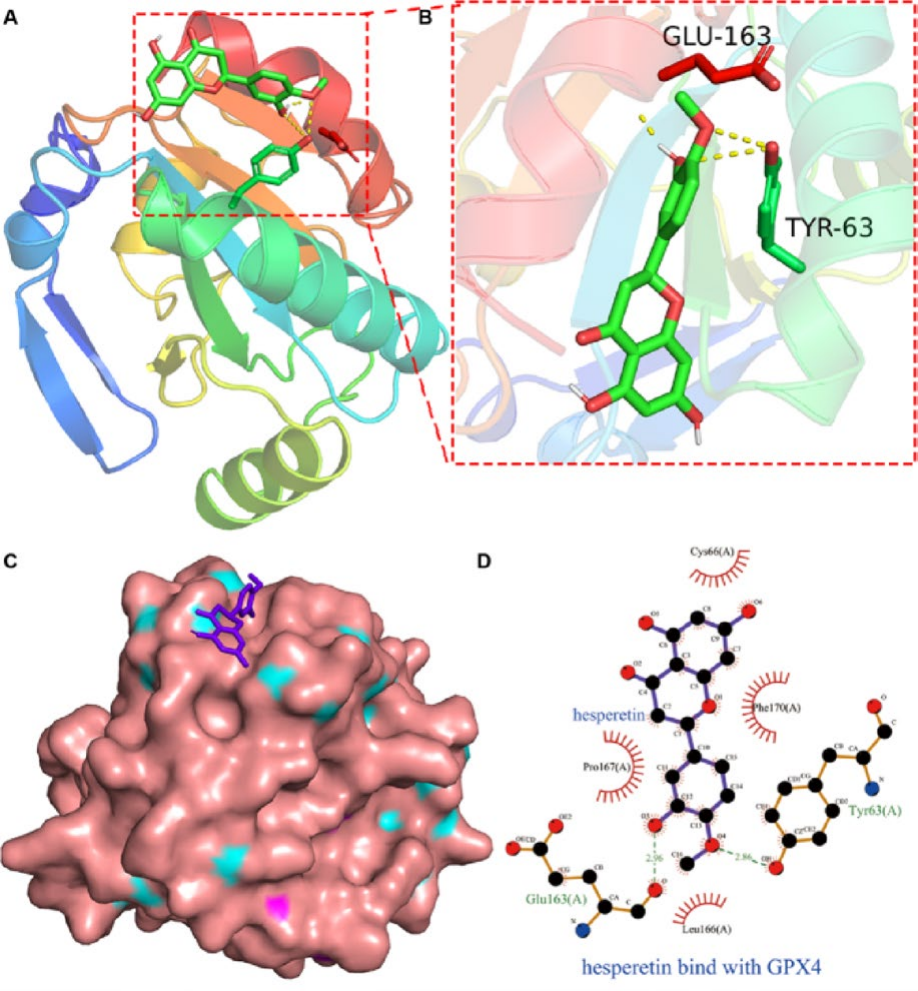
Hesperetin-GPX4 molecular docking diagram.

In GPX4 (PDB ID: 7u4j), hesperetin compound forms hydrogen bond (Fig. 5A, B) with amino acid residues Tyr-63 (2.86) and Glu-163 (2.96) of the protein and occupies the active cavity position of the protein (Reference 36,423,641, Fig. 5C), and amino acid residues CYS-66, LEU-166, PRO-167. PHE-170 forms a hydrophobic interaction) (Fig. 5D), and its free energy of binding to the protein is − 5.8 kcal/mol, indicating a potential interaction between hesperetin and GPX4 protein.

## Discussion

Cerebral ischemia–reperfusion injury damages neurons in multiple ways, including neuronal oxidative stress, inflammatory response, neuronal apoptosis, and neuronal ferroptosis, leading to expansion of cerebral infarct size. In the present study, we evaluated the protective effects of hesperetin against cerebral ischemia–reperfusion injury from multiple perspectives. At the cellular level, hesperetin significantly increased cell viability after ischemia–reperfusion in a dose-dependent manner, which was attributed to hesperetin’s inhibition of apoptosis induced by oxidative stress in cells after ischemia-reperfusion^18^. In vivo metabolism tightly controls ROS production, and large amounts of ROS are produced during postischemic reperfusion, triggering oxidative damage, which in turn triggers lipid peroxidation and mitochondrial membrane potential changes^19,20^. In the present experiments, we detected changes in the content of the products of interest in a cellular model of OGD/R. By HSP treatment, the ROS content of the cells after OGD/R was significantly reduced, and the cellular MDA content, GSH content, and mitochondrial membrane potential were all reversed. HIF1α is an oxygen-sensitive transcription factor and a key signaling molecule for oxidative responses, and HIF1α levels are upregulated after ischemia-reperfusion^21,22^. Ferroptosis is defined as a form of iron-dependent cell death driven by lipid peroxidation, and the GSH-GPX4 axis is thought to be the major pathway of ferroptosis^20,23^. In addition, ACSL4 is a key promoter of ferroptosis. It is activated by catalyzing the oxidation of polyunsaturated fatty acids (PUFAs), thereby providing synthetic substrates for PUFA-rich phospholipids (PUFA-PLs) in the cell membrane. These substrates serve as direct precursors for lipid peroxidation reactions, and their accumulation directly determines the cell’s susceptibility to ferroptosis^24^. GPX4 acts as a central inhibitor of ferroptosis. It is the only enzyme capable of specifically clearing phospholipid hydroperoxides, with the assistance of reducing equivalents provided by GSH, it reduces toxic lipid peroxides to non-toxic lipols, thereby blocking the cascade of oxidative damage^25,26^. When GPX4 activity is lost due to GSH depletion or downregulation of GPX4 expression, cells are unable to clear the lipid peroxide substrates abundantly supplied by ACSL4. The combined effect of these two factors leads to a rapid accumulation of lipid peroxide products within the cell, ultimately triggering ferroptosis^27^. In our study, HSP inhibited lipid peroxidation by upregulating GSH levels and GPX4 expression, while downregulating ACSL4 expression, in cells subjected to OGD/R, thereby effectively suppressing ferroptosis. In addition, the reduction of intracellular iron ion content further corroborated the ferroptosis inhibitory effect of HSP. We found the same changes in the brain tissue of MCAO mice and visually evaluated the protective effect of HSP on the brain tissue of mice after MCAO by TTC staining of brain tissue sections. In our study, we also found that brain tissue genes in MCAO mice were mainly enriched in genes related to inflammation, apoptosis, oxidative stress, and ferroptosis. These results suggest that HSP inhibits postischemic reperfusion injury in brain tissue mainly by inhibiting neuronal ferroptosis. However, our current study has some limitations that need to be further explored. In addition, there are fewer studies on HSP on cerebral ischemia–reperfusion injury, which has potential research value and application potential.

## Conclusion

This study demonstrates that HSP exerts a significant protective effect on neurons following OGD/R. Its mechanism of action involves inhibiting neuronal ferroptosis by upregulating GSH and GPX4 and downregulating ACSL4, thereby exerting antioxidant and anti-apoptotic effects. This provides a new direction for research and clinical treatment of OGD/R.

## Materials and methods

### Detection of cell activity by CCK-8 method

PC12 cells in logarithmic phase were prepared into cell suspension with the density of 5 × 10^**4**^/mL. Two 96-well plates were selected, one plate being used to set the blank group and the control group, and the other plate being used to set the model group and the administration group. Three multiple wells were set in each group, and 100 μl cell suspension was added into each well. After the cells were shaken uniformly by the cross shaking method, they were placed in an incubator at 37 °C and 5%CO_**2**_ for culture. After the cells grew to about 80%, the complete medium in the well plate was removed, and the serum-free and non-antibody-free sugar-containing medium was added into the blank group and the control group and put back to the incubator at 37 °C and 5% CO_**2**_. The model group and the administration group were added with sugar-free medium, and then placed in the three-gas incubator set at 37 °C, 5%CO_**2**_ and 1% O_**2**_ for 4 h to construct cell OGD model. After the treatment, the sugar-free medium was removed, and the serum-free and non-anti sugar-containing medium was added to the model group. The hesperetin solution with the concentration of 25 μM, 50 μM and 100 μM was added to the administration group for treatment under the condition of 37 °C and 5% CO_**2**_ for 24 h. After the treatment was completed, the cell viability was measured using Cell Counting Kit-8.

### Western blotting

PC12 cells in logarithmic phase were prepared into cell suspension with the density of 1 × 10^6^/mL. Two six-well plates were selected, with one plate set as the control group and the other plate as the model group and administration group. 1 mL of cell suspension and 1 mL of complete culture medium were added into each well. After the cells were shaken uniformly by the cross shaking method, they were placed in an incubator at 37°C and 5% CO_2_ for culture. After the cells grew to about 80%, the complete medium in the well plate was removed, and the serum-free and non-antibody-free sugar-containing medium was added into the blank group and the control group and put back to the incubator at 37 °C and 5% CO_**2**_. The model group and the administration group were added with sugar-free medium, and then placed in the three-gas incubator set at 37 °C, 5% CO_2_ and 1% O_2_ for 4 h to construct cell OGD model. After the treatment, the sugar-free medium was removed, and the serum-free and non-anti sugar-containing medium was added to the model group. The HSP solution with the concentration of 25 μM, 50 μM and 100 μM was added to the administration group for treatment under the condition of 37 °C and 5% CO_2_ for 24 h. Extracting cell protein, performing agar gel electrophoresis, cutting PVDF membranes with corresponding sizes after electrophoresis is completed, carrying out membrane transfer after soaking in methanol, taking out the PVDF membranes after the membrane transfer is completed, sealing by using 5% defatted milk, washing the membranes by using TBST for three times, 5 min each time, and incubating the primary antibody overnight under the condition of 4 °C, washing the membranes by using TBST for three times, 10 min each time after the incubation of the primary antibody is completed, incubating the secondary antibody for 1 h at normal temperature, and washing the membranes by using TBST for three times, 10 min each time after the incubation of the secondary antibody is completed, and then developing by using ECL chemiluminescent substrate.

### Detection of apoptosis by flow cytometry

PC12 cells in logarithmic phase were prepared into cell suspension with the density of 3 × 10^5^/mL. Two six-well plates were selected, with one plate set as the control group and the other plate as the model group and administration group. 1 mL of cell suspension and 1 mL complete medium were added into each well. After the cells were shaken uniformly by the cross shaking method, they were placed in an incubator at 37°C and 5% CO_2_ for culture. After the cells grew to about 80%, the complete medium in the well plate was removed, and the serum-free and non-antibody-free sugar-containing medium was added into the blank group and the control group and put back to the incubator. The model group and the administration group were added with sugar-free medium, and then placed in the three-gas incubator set at 37 °C, 5% CO_2_ and 1% O_2_ for 4 h to construct cell OGD model. After the treatment, the sugar-free medium was removed, and the serum-free and non-anti sugar-containing medium was added to the model group. The HSP solution with the concentration of 25 μM, 50 μM and 100 μM was added to the administration group for treatment under the condition of 37 °C and 5% CO_2_ for 24 h. After the treatment, each group of cells were prepared into cell suspension, and then the cells were stained according to the Annexin V-FITC/PI Apoptosis Detection Kit instructions. After the staining, the cells were detected on the computer by flow cytometry, and the data were analyzed by FLOWJO software.

### Detection of MDA, LDH and GSH content in cell

PC12 cells in the logarithmic phase were inoculated into 96-well cell culture plates. When the cell density reached about 80%, the complete medium was removed and washed once with PBS. Serum-free medium without any anti-sugar was added to the control group. Sugar-free medium was added to the model group and the administration group. Then, the cells were placed in a three-gas incubator set at 37 °C, 5% CO_2_ and 1% O_2_ for treatment for 4 h to construct an OGD model of cells. After the treatment, the sugar-free medium was removed, and the serum-free and non-anti sugar-containing medium was added to the model group. The HSP solution with the concentration of 25 μM, 50 μM and 100 μM was added to the administration group for treatment under the condition of 37 °C and 5% CO_2_ for 24 h. Then that cytotoxicity is detected according to the instruction of the corresponding detection kit, and finally the cell death rate is calculated according to the OD value.

### Cellular iron ion assay

Control, OGD/R, HSP25, HSP50 and HSP100 treated PC12 cells were cultured separately. About 1 × 10^6^ cells were taken from the collected cells, and then the absorbance at 593 nm was measured after labeling using Cell Total Iron Colorimetric Assay Kit (Elabscience).

### Animal model construct

Male mice (C57BL/6, 20-25g, 6–8 weeks old) were obtained from Mudanjiang Medical University Laboratory Animal Center, Mudanjiang, China, and housed under specific pathogen-free conditions on a 12 h/12 h light/dark cycle. All animal experiments were approved by the Laboratory Animal Welfare and Ethics Committee of Mudanjiang Medical University, and were conducted in accordance with the Guidelines for the Care and Use of Laboratory Animals. The license of Mudanjiang Medical University involved the application of animal experiments (IACUC-20240606–136). Mice were anesthetized with 2% isoflurane. After the midline incision of the neck, the right common carotid artery, external carotid artery and internal carotid artery were removed. A silicone-coated distal MCAO monofilament was inserted through the ECA into the internal carotid artery (ica), resulting in a flow occlusion of the middle cerebral artery (MCA). The MCAO monofilament was removed for reperfusion after 1 h. Suture the incision and let the mice recover after keeping warm under a heating lamp. Mice were weighed at different time points after MCAO. Mice were randomly divided into the following experimental groups before operation: Control, MCAO, and HSP (n = 15). Three were used for TTC staining, three for ELISA testing, three for LSCI imaging, three for Western blotting, and three for transcriptomic sequencing. First, HSP was dissolved in DMSO, and then diluted with PBS to the final concentration of 10 mg/mL. On the 2nd and 4th day after MCAO, administration (HSP of 30 mg/kg) was given through the tail vein immediately. The establishment of control group, except occlusion, is consistent with the treatment results of MCAO group. Compared with other experimental groups, MCAO group was injected with the same amount of PBS.

### Longa test and rotation test

Neurobehavioral dysfunction caused by ischemic stroke was tested by Longa test (n = 15). The scale is as follows: 0: normal, no neurological deficit; 1–2: unable to fully extend forelimbs or hind limbs, with mild neurological deficit; 3–4: straight walking failure, hemiplegia, moderate neurological deficit; 5–6: Turn right (hemiplegic side), with severe neurological deficit; 7–8 points: unable to walk autonomously and lose consciousness. Balance and motor coordination were evaluated by rotation test (n = 15). Mice were trained for 2 days before MCAO, and gradually accelerated from 4 to 20 rpm within 5 min. When the mice fell from the sight bar in three experiments, the average time of falling latency was measured.

### TTC staining

Mice were sacrificed under deep anesthesia on the 3rd day (n = 3 cases in each group). The brain was cut into 2 mm thick slices and incubated in 2% 2,3,5- triphenyltetrammonium chloride (TTC). The slices were incubated in TTC solution at 37 °C for 20 min. The infarct area was measured by Image J analysis software.

### Determination of IL-6 and TNF-α levels in mice brain tissue

Brain tissue was collected from normal, MCAO, and HSP treated mice (n = 3). RIPA lysis buffer was added to the brain tissue, which was then homogenized and centrifuged; the supernatant was collected and set aside. The mouse IL-6 and TNF-α assay kits were brought to room temperature. Sampling, incubation, and detection were performed according to the kit instructions.

#### Transcriptomic sequencing

The rRNA was removed from the total RNA via the Zymo-Seq RiboFree Total RNA Library Kit, and then the RNA was randomly disrupted via ionic interruption with divalent cations. cDNA was synthesized using RNA templates and random oligonucleotides as primers. cDNA was purified from double-stranded cDNA, followed by double-end repair and introduction of the “A” base at the 3’ end and ligation to the sequencing junction. The cDNA was screened for cDNAs of 400–500 bp via AMPure XP beads and amplified via PCR, and the PCR products were subsequently purified again via AMPure XP beads to obtain the final library. The libraries were analyzed via an Agilent 2100 Bioanalyzer (Agilent, 2100) and an Agilent High Sensitivity DNA Kit (Agilent, 5067–4626). The total library concentration was detected via Pico green (Quant-iT PicoGreen dsDNA Assay Kit, Invitrogen, P7589), and the concentration of the validated library was quantified via q-PCR (Thermo Scientific StepOnePlus Real-time DNA Assay Kit, P7589). Multiplexed DNA libraries were homogenized and mixed in equal volumes. The mixed libraries were gradually diluted and quantified and then sequenced in PE150 mode on an Illumina sequencer. DEGs with adjusted *p*-values < 0.05 and log_2_FC >|1| were selected for downstream representation analysis. Using GenesCloud for visualization.

#### Statistical analysis

The data were analyzed statistically and are presented as the mean ± standard deviation (SD). The difference between groups was statistically significant according to a two-tailed t test. one-way ANOVA with Tukey’s post-hoc test was used for multiple-group comparisons. *p* values greater than or equal to 0.05 were considered nonsignificant at the 95% confidence level. *P < 0.05, **P < 0.01, ***P < 0.001, ****P < 0.0001. # *P* < 0.05, ##*P* < 0.05, ###*P* < 0.05, ####*P* < 0.0001. All statistical analyses were performed via GraphPad Prism 8.0.1.

## Data Availability

The RNA-seq data in this study and any additional information required to reanalyze the data reported in this paper is available from the correspondence author upon request.
